# The microneme proteins CTRP and SOAP are not essential for *Plasmodium berghei *ookinete to oocyst transformation *in vitro *in a cell free system

**DOI:** 10.1186/1475-2875-7-82

**Published:** 2008-05-19

**Authors:** Adéla Nacer, Ann Underhill, Hilary Hurd

**Affiliations:** 1 Centre for Applied Entomology and Parasitology, Institute of Science and Technology in Medicine, School of Life Sciences, Keele University, Staffordshire, ST5 5BG, UK

## Abstract

**Background:**

Two *Plasmodium berghei *ookinete micronemal proteins, circumsporozoite and TRAP related protein (CTRP) and secreted ookinete adhesive protein (SOAP) both interact with the basal lamina component laminin. Following gene disruption studies it has been proposed that, apart from their role in motility, these proteins may be required for interactions leading to ookinete-to-oocyst transformation.

**Methods:**

CTRP and SOAP null mutant *P. berghei *ookinetes were compared to *P. berghei *ANKA wild-type for their ability to transform and grow *in vitro*. To confirm *in vitro *findings for *P*. *berghei *CTRP-KO ookinetes were injected into the haemocoel of *An*opheles *gambiae *female mosquitoes.

**Results:**

Transformation, growth, and viability were comparable for the gene disrupted and wild-type parasites. *P*. *berghei *CTRP-KO ookinetes were able to transform into oocysts in the haemocoel of *An*. *gambiae *mosquitoes.

**Conclusion:**

Neither CTRP nor SOAP is required for parasite transformation *in vitro*. By-passing the midgut lumen allows for the transformation of *P*. *berghei *CTRP-KO ookinetes suggesting that it is not required for transformation *in vivo*.

## Background

Ever since the early 1900s, an appreciation of the role that anopheline mosquitoes play in the transmission of malaria [1] has directed disease control efforts towards the vectors. Many campaigns relied upon insecticide usage and, latterly, the use of insecticide treated bed nets is proving effective [2]. However, attendant concerns regarding the development of insecticide resistance [2,3] make it imperative that new tools are developed to inhibit malaria transmission. This will be best achieved by gaining and applying a better understanding of vector-parasite interactions.

The sporogonic stages which occur in the mosquito vector begin when a mosquito ingests infected blood containing gametocytes. Within approximately 18 hours, gametogenesis, fertilization, and the transformation of the zygote to a motile ookinete have occurred. Ookinetes migrate out of the blood bolus, through the peritrophic matrix and invade and transit through the midgut epithelium cells, transforming into oocysts beneath the basal lamina 20–30 hours post-bloodfeeding. The initial period post-infection is critical for the malaria parasites as losses of up to 100,000 fold occur, dependant upon parasite/vector combinations [4-8].

Within recent years, knowledge of the ookinete at the molecular level has burgeoned and many proteins have been shown to be involved in midgut invasion. These include several with functions associated with motility and/or invasion, such as the major surface proteins P25/P28 [9-11], secreted ookinete adhesive protein (SOAP) [12], von Willebrand Factor A Domain-related protein (WARP) [13], circumsporozoite and TRAP-related protein (CTRP) [14-19], cell-traversal protein (CelTOS) [20], calcium-dependent protein kinase 3 (CDPK3) [21,22], the guanylate cyclase, PbGCβ [23], membrane-attack ookinete protein (MOAP) [24,25], and *Plasmodium *perforin-like proteins [26]. The rodent malaria, *Plasmodium berghei*, has proven to be a useful model with which to investigate the function of these proteins and targeted gene disruption has assisted these studies.

The micronemal protein CTRP, first described in *Plasmodium falciparum *by Trottein *et al *[17], has structural homologues in *P. berghei *[14,18], *Plasmodium vivax, Plasmodium knowlesi, Plasmodium gallinaceum, and Plasmodium yoelii *[27]. CTRP contains two types of adhesive domains (von Willebrand factor type A-related and thrombospondin type 1-related) and belongs to a family of proteins located in the micronemes of several genera of apicomplexan parasites [14,28]. It is expressed in *P. berghei *ookinetes from 10 hours post-fertilization and expression continues for at least 24 hours [18]. Using immuno-electron microscopy, Limviroj and colleagues [15] localized PbCTRP to the ookinete micronemes and detected it at the site of contact with the basal lamina; suggesting that the junction so formed may induce the ookinete to stop and begin to transform into an oocyst.

Targeted disruption of CTRP has demonstrated that, although ookinetes form, transgenic parasites fail to develop into oocyst *in vivo*, consequently blocking mosquito transmission of these parasites [14,16,19]. These studies further demonstrated that CTRP is involved in ookinete locomotion [14]. CTRP has also been shown to bind to laminin and collagen IV, major components of the basal lamina, prompting further suggestions of a putative role in the transformation of ookinete to oocyst *via *involvement in a signal transduction pathway [29,30].

SOAP, another microneme protein, is unique to the genus *Plasmodium*. It is expressed in ookinetes and young oocysts and asexual stages but has not been reported in gametocytes of *P. berghei *and thus has a similar transcriptional pattern in the mosquito to PbCTRP [12,31]. It is a cysteine-rich, secreted protein not present on the parasite surface. Dessens and colleagues [12] showed that disrupting the SOAP gene resulted in null mutant parasites that were impaired in their ability to invade the mosquito midgut wall and form oocysts. They further showed that this 21-kDa protein also interacts with laminin in a yeast two-hybrid system and suggested that EGF-like domains of laminin gamma 1 interact with SOAP sequence domains that are homologous to laminin EGF-like domains [12]. SOAP was thus thought to play an important role during ookinete-to-oocyst development in mosquitoes [12]. Interestingly, they also showed that PbSOAP-KO ookinetes invaded co-cultured mosquito cells and transformed into oocysts therein at a more efficient rate than wild-type ookinetes; a finding that is at odds with their identification of SOAP as a key molecule for ookinete-to-oocyst differentiation in the mosquito [12].

PbCRTP and PbSOAP null mutants, grown in an axenic culture system [32], were used to determine the role, if any, of these microneme proteins in the transformation of ookinetes into oocysts. Ookinetes were cultured in the presence or absence of a basal lamina substitute. The results show that neither microneme protein is essential for transformation as ookinetes from both parasite null mutants are able to transform into oocysts *via *the transitional *took *stage recently described by Carter *et al *[32].

## Methods

### Parasites and ookinete cultures

Experiments were performed using approved protocols in accordance with the UK. Animals (Scientific Procedures) Act 1986. Male CD mice were treated with phenylhydrazine two days prior to infection with either *P. berghei *ANKA (clone 2.34; wild-type), *P. berghei *CTRP-KO [14], or *P. berghei *SOAP-KO [12] by the inoculation of parasites obtained from a donor mouse between the 2^nd ^and 6^th ^passage from cryopreserved stock.

Blood from infected mice was collected by cardiac puncture into a heparinized syringe two days post-infection, after the confirmation of an infection by Giemsa staining of thin blood smears. Blood was diluted 1:10 into RPMI-1640 medium containing 10% foetal calf serum (FCS), 14.7 mM sodium bicarbonate, 0.367 mM hypoxanthine, 1000 U/ml penicillin, and 1 mg/ml streptomycin (pH 8.4) prior to incubation for 18 h at 19°C as detailed in [33,34].

Ookinetes were harvested using a MidiMacs LS magnetic column as described previously [32,35]. Briefly, following centrifugation at 648 g for 5 min all but 20% of the medium was removed and the blood mixture was passed through a magnetic column. Following several column washes, ookinetes were eluted with 3 ml of oocyst medium (supplemented Schneider's insect cell culture medium).

*Plasmodium berghei *wild-type (WT), PbCTRP-KO or PbSOAP-KO ookinetes were immediately seeded into 8-well LabTek™ chamber slides at a density of approximately 1 × 10^4 ^in 400 μl of oocyst medium (see [32] for medium details). For each experiment, ookinetes were seeded in chamber slides either previously coated with 80 μl Matrigel™ or with no coating. In order to minimize parasite loss from the wells, medium was not removed during culture. However, to counter the effect of evaporation, 100 μl of fresh medium was added to each well every 48 or 72 h post-culture. Triplicate wells were assessed at each time point and the experiment was repeated three times.

### Demonstration of morphological changes during transformation

The presence of a transitional stage between ookinete and oocyst in *P. berghei *ANKA WT, called the *took*, was recently reported [32]. To demonstrate clearly the presence of a *took *stage during the development of oocysts of the null mutants, cultures were collected 21 h post-culture in oocyst medium. Parasites were fixed in 1:1 methanol: acetone, washed and labelled with an anti-P28 monoclonal antibody derived from a hybridoma cell line maintained in the laboratory. Following a 30 min incubation the slides were washed and incubated for 30 min with a goat anti-mouse fluorescein isothiocyanate-conjugated secondary antibody [32]. The slides were washed and mounted in VectaShield (Vector Laboratories) containing 4', 6-diamidino-2-phenyl indole (DAPI) before viewing samples with a Leica DM IRB Fluorescence Microscope (DAPI excitation/emission: 340–380 nm/425 nm; FITC: 450–490 nm/535 nm). Appropriate controls were included for each experiment.

### Assessment of parasite transformation and growth

Ookinetes have a propensity to clump together in culture, so well contents were mixed vigorously by pipetting to ensure even distribution within the well prior to counting ookinetes/oocysts. Measurements and counts were conducted on days 1, 3 and 5 post-culture for wells that were not coated and day 1 for wells coated in Matrigel. Parasites were counted in alternate fields of view along a cross representing two diameters of the well, at a magnification of 630×, viewed under phase contrast. In all, 30 fields of view were examined per well (3.58% of the whole well). In order to assess transformation of ookinetes to oocysts, the proportion of *P. berghei *WT, PbCTRP-KO or PbSOAP-KO ookinetes, *tooks *and oocysts was calculated for each culture condition at each time point. Only parasites that were fully rounded, translucent, with pigment visible and a diameter greater than 5 micrometers were regarded as having transformed into oocysts [32].

### Oocyst viability

Parasites were collected by centrifugation for 1.5 min at 2,000 g. The pellet was resuspended in 5 μl of PBS to which 5 μl of 0.1% Erythrosin B was added to assess parasite viability. This was immediately spotted onto a microscope slide, a coverslip applied and a minimum of 100 oocysts and any remaining ookinetes and *tooks *were examined at magnification of 400× and scored as viable or having a compromised membrane. Three repeat experiments were performed in which triplicate wells were assessed on each of the days chosen for observation.

### Injection of *Anopheles gambiae *mosquitoes with *P. berghei *CTRP KO ookinetes

Unlike PbSOAP-KO, for which it has been reported that between 15–40% of ookinetes transform into oocysts *in vivo *[12], no PbCTRP-KO oocysts have been observed *in vivo *[14,16,19]. In order to confirm the lack of a role of CTRP in transformation, *in vitro *ookinete cultures were injected into the haemocoel of 4 day old *An. gambiae *KIL mosquitoes. The preparation and injection protocols were adapted from Paskewtiz and Shi [36]. Briefly, PbCTRP-KO ookinetes were cultured for 18 h at 19°C in ookinete medium. Cultures were centrifuged at 648 g for 5 min at 4°C. The majority of red blood cells were removed by resuspending the culture pellet in 20 volumes of 0.17 M ammonium chloride and incubating for 10 min on ice. All subsequent steps were performed at 4°C. An equal volume of PBS was added to stop red blood cell lysis and cultures were immediately centrifuged. The pellet was washed once in 40 volumes of PBS, resuspended in 1 ml of PBS and counted on a haemocytometer.

Fifty ookinetes in 69 nl of PBS were injected per mosquito with a Nanoject II automatic nanoliter injector (Drummond Scientific Company, USA). A total of 50 mosquitoes were injected and maintained at 20°C for seven days prior to dissection. Mosquitoes were dissected in PBS and the contents of the abdomen were immediately stained in 0.05% mercurochrome. Tissue was viewed on a Leica DM IRB inverted microscope and images were acquired with a Leica DC 300F digital camera (Leica Microsystems, Germany).

### Statistical analysis

All data sets were analysed using the statistical software package Minitab^® ^Release 14 (Minitab, Inc.). Where necessary, data were normalized with an arcsine transformation prior to analysis with a General Linear Model (GLM).

## Results and discussion

### Transformation of CTRP- and SOAP-KO ookinetes to oocysts

**Figure 1:**
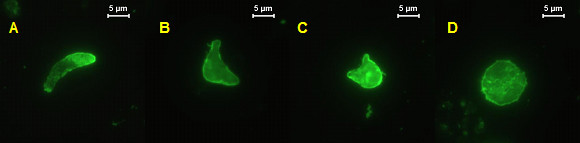
**Transformation of *P. berghei *CTRP-KO ookinetes *via *a *took *stage**. Ookinetes were cultured in oocyst medium for 21 h and labelled for the major ookinete surface protein, P28, by indirect immunofluoresence assay. A) mature ookinete; (B, C) *tooks*; (D) oocyst.

It has been proposed that SOAP and CTRP may play a role in ookinete-to-oocyst transformation *in vivo *as the null mutants for either gene produce very few, or no oocysts respectively [12,14,19]. Moreover, both of these molecules have been reported to bind to laminin and it has been proposed that interactions between SOAP and/or CTRP with the basal lamina, and laminin in particular, may trigger transformation [12,30]. However, it was found that when *P. berghei *CTRP- and SOAP-KO ookinetes were cultured *in vitro*, plus or minus Matrigel (a source of laminin), the same proportion transformed to oocysts as did the wild-type. Furthermore, transformation proceeded via a *took *stage (Figure 1) as recently described by Carter *et al *[32].

The percentage of transformed ookinetes (i.e. oocysts) was calculated on days 1, 3, and 5 post-culture after counting the number of ookinetes, *tooks *and oocysts per well. Over all conditions, a mean of 93% (SE ± 1.33) of ookinetes had transformed into oocysts by day 1. There was no significant difference in the percentage of ookinete to oocyst transformations between parasites lines (GLM, *F*_(*2,1,2,2*) _= 1.64, *P *= 0.213) or the presence or absence of a basal lamina substitute (GLM, *F*_(*2,1,2,2*) _= 0.60, *P *= 0.446) (Table 1). As the majority of parasites had transformed by day 1 and no differences were observed between culture conditions, data for day 3 and 5 was only collected from wells that were not coated with Matrigel. For all parasite genotypes, nearly 100% of ookinetes had transformed by day 5 (Table 1).

**Table 1:** Ookinete to oocyst transformation of *P. berghei *WT, PbCTRP-KO, and PbSOAP-KO parasites.

	**Mean % transformation**		
	Day 1	Day 3	Day 5

WT -MAT	92.68 (± 1.51)	99.73 (± 0.17)	99.94 (± 0.06)
WT +MAT	96.27 (± 0.86)		
CTRP-KO -MAT	96.76 (± 0.78)	99.70 (± 0.22)	100.00 (± 0.00)
CTRP-KO +MAT	98.85 (± 0.45)		
SOAP-KO -MAT	87.05 (± 5.38)	99.13 (± 0.43)	100.00 (± 0.00)
SOAP-KO +MAT	89.21 (± 4.89)		

Ookinetes from each of the clones were seeded in the absence of Matrigel (-MAT) or with Matrigel (+MAT). Transformation was assessed on days 1, 3, and 5 post-culture. The figures show the mean percentage of transformed ookinetes (± standard error of the mean).

The large percentage of ookinete-to-oocyst conversion in these experiments was surprising as it has previously been reported that over 30% of ookinetes cultured *in vitro *are dying by an apoptosis-like mechanism by 24 h post-culture [37]. This suggests that ookinetes harvested at 18 h post-culture and immediately placed in oocyst medium, which supports transformation and growth (see [32]), may be rescued from triggers that induce cell death. It was previously observed that the proportion of ookinetes displaying markers typical of apoptotic cells was under 10% after 12 h in culture [37]. Current findings confirm this observation, but also show that the proportion of dying or dead ookinetes varies considerably between cultures, possibly as a result of factors from the murine host (Hurd, Ali and Arambage, personal observations).

**Figure 2:**
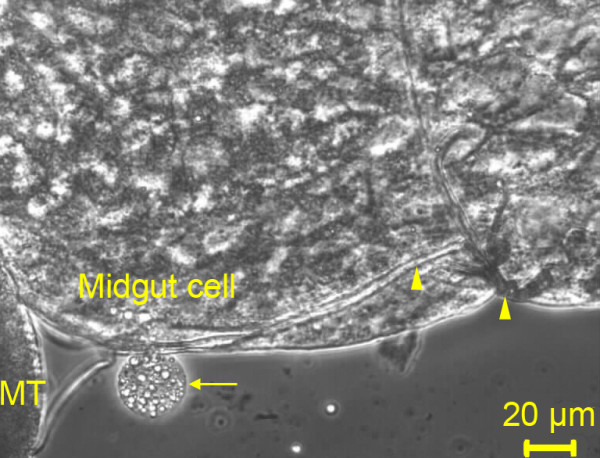
**Transformation of PbCTRP-KO ookinetes in *Anopheles gambiae***. Phase contrast image of a PbCTRP-KO oocyst (arrow) attached to the midgut of *An. gambiae *KIL mosquito. The trachea (arrow heads) and Malpighian tubules (MT) are also shown. *Plasmodium berghei *CTRP-KO ookinetes were injected 18 h post-culture into the haemocoel of 4-day old *An. gambiae *KIL females and allowed to develop for 7 days prior to mosquito dissection.

Of the 50 mosquitoes injected with *P*. *berghei *CTRP-KO ookinetes only four survived to day 7 post-injection. *P*. *berghei *CTRP-KO oocysts were observed in all four mosquitoes. Thus PbCTRP-KO ookinetes are able to transform into oocysts when injected into the haemocoel of 4-day old female *An*. *gambiae *KIL mosquitoes. Qualitative observations were made of the infections as it is difficult to quantify oocysts as they can develop anywhere in the haemocoel [36]. Upon dissection the majority of oocysts recovered were observed to be floating in the haemolymph. However, oocysts were also found bound to the midgut (Figure 2) and the Malpighian tubules.

These findings confirmed suggestions that both PbCTRP and PbSOAP null mutants have retained their full potential to develop into oocysts. Both microneme proteins have been implicated in ookinete motility [12,14,16,19,27,38] and findings presented here suggest that the barrier to further development *in vivo *must be caused by the necessity to migrate out of the blood bolus, traverse the peritrophic matrix and invade the midgut epithelium. Dessens *et al *[12] also observed the transformation of PbSOAP-KO parasites *in vitro*, but surprisingly, in their system ookinetes invaded and developed inside *Aedes aegypti *Mos20 cells. The axenic culture system used here also shows that cell invasion is not a prerequisite for transformation. Interestingly, Hirai *et al *[23] recently reported that disruption of the gene encoding PbGCβ also resulted in normal ookinetes that do not produce oocysts *in vivo*. Here too these transgenic ookinetes transformed into oocysts in an *in vitro *system based on Al-Olayan *et al *[37] and ookinete motility was shown to be defective.

### Interactions with the basal lamina

Transformation rates of null mutants and wild-type *P. berghei *were the same in the presence or absence of Matrigel in this study (Table 1). These results suggest that the binding of either one of these micronemal proteins to basal lamina components is not a requirement for transformation. Indeed, the study also confirms that interactions between basal lamina components and any ookinete molecule are unnecessary to initiate conversion to oocysts [32]. It has been suggested that the mosquito midgut basal lamina plays an essential role in the development of *Plasmodium *oocysts [39]. Gene silencing technology resulting in the depletion of mosquito laminin greatly reduced oocyst numbers developing *in vivo *[39] and interactions between laminin and microneme and/or surface proteins support this suggestion [12,29]. If this idea is correct then, as it is clearly not involved in triggering transformation or supporting very early growth, it may perform a function as a protective coat, disguising the oocyst from the mosquito defence system [39,40] or just serve to bind the ookinete in place between the midgut basolateral plasma membrane and the basal lamina, thus preventing the ookinetes from migrating into the haemocoel, as do most insect parasites.

The identification of the molecular composition of the mosquito midgut is rapidly advancing. The major components of the basal lamina, collagen IV and laminin, have been characterized in *An*. *gambiae *[41,42], as has beta integrin, a receptor for laminin and collagen IV[43]. The latter has been implicated in ookinete invasion of the midgut and may have a role in facilitating putative interactions between ookinetes and laminin or *vice versa *[43]. However, it now appears that these molecules are more likely to be involved in later phases of oocyst development such as growth, maturation, and sporozoites formation.

### Growth and viability

**Figure 3:**
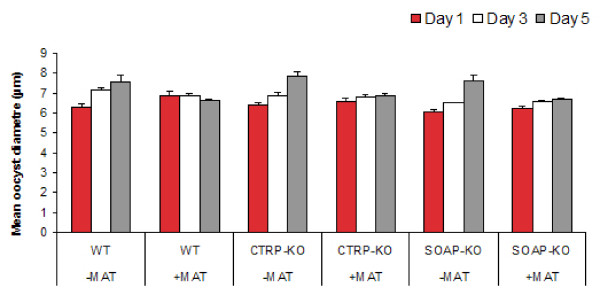
**The effect of basal lamina components on the growth of *P. berghei *wild-type, PbCTRP-KO, and PbSOAP-KO oocysts**. Three clones of *Plasmodium berghei *were cultured in the presence or absence of Matrigel. -MAT = no Matrigel; +MAT = with Matrigel; WT = *P. berghei *ANKA clone 2.34; CTRP-KO = *P. berghei *CTRP-KO; SOAP-KO = *P. berghei *SOAP-KO. No differences were observed in the mean size of oocysts cultured in the presence or absence of Matrigel or between parasite clones.

**Figure 4:**
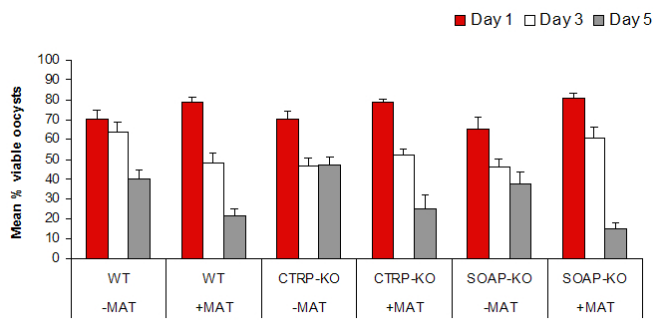
**The effect of basal lamina components on the viability of *P. berghei *wild-type, PbCTRP-KO, and PbSOAP-KO oocysts**. Three clones of *Plasmodium berghei *were cultured in the presence or absence of Matrigel. -MAT = no Matrigel; +MAT = with Matrigel; WT = *P. berghei *ANKA clone 2.34; CTRP-KO = *P. berghei *CTRP-KO; SOAP-KO = *P. berghei *SOAP-KO. There was a significant reduction in the number of viable oocysts with increasing time of culture (GLM: F_(2,1,2,2) _= 117.32, *P *< 0.001).

In addition to transforming into oocysts, PbCRTP-KO and PbSOAP-KO parasites were able to grow in culture at a comparable rate to *P. berghei *WT and, here too, no significant differences were found between the presence and absence of the basal lamina substitute (Figure 3). Over the five day period there were significant increases in parasite sizes. Tukey's pairwise comparison day 1 vs day 3 (*T *= 4.946, *P *< 0.001), and day 3 and 5 (T = 3.195, *P *< 0.01) (Figure 3). However a significant decrease in the number of viable oocysts between each of the time points was observed (GLM: F_(2,1,2,2) _= 117.32, *P *< 0.001), but with no significant difference between parasite genotype (GLM: F_(2,1,2,2) _= 0.70, *P *= 0.497) or the presence or absence of Matrigel (GLM: F_(2,1,2,2) _= 1.85, *P *= 0.175) (Figure 4). Although not statistically significant, fewer parasites were viable when incubated with Matrigel at day 5. This could be caused by additional gentamycin, present in Matrigel as well as in the oocyst culture medium.

## Conclusion

The microneme proteins SOAP and CTRP are not involved in ookinete to oocyst transformation or early growth *in vitro*. Furthermore, by by-passing the need to invade the midgut, PbCTRP-KO ookinetes are capable of transforming into oocysts in the haemocoel of *An*. *gambiae *mosquitoes. These results suggest that the role of these micronemal proteins may be restricted to motility and/or invasion.

In order to find adequate targets for transmission blocking strategies it is necessary to gain a better understanding of the proteins expressed during the malaria parasite sporogonic stages and their influence on parasite development. The technique of gene disruption is an extremely useful tool in this quest and it is suggested that, in additional to *in vivo *studies to determine gene function, an *in vitro *culture system will facilitate these investigations.

## Authors' contributions

AN and HH prepared the manuscript, AN performed the experiments with assistance from AU. HH conceived of the project. Both authors have read and approved the final version of the manuscript.
